# Cognitive Disengagement Syndrome Symptoms and ADHD Dimensions in Relation To Children’s Daily Life Executive Functioning Deficits

**DOI:** 10.1007/s10802-026-01436-z

**Published:** 2026-02-19

**Authors:** Melissa C. Miller, Emmarald Jean-Francois, Kate E. Hembree, James L. Peugh, Jeffery N. Epstein, Leanne Tamm, Stephen P. Becker

**Affiliations:** 1 Division of Behavioral Medicine and Clinical Psychology, Cincinnati Children’s Hospital Medical Center, 3333 Burnet Avenue, MLC 10006, Cincinnati, Ohio 45229-3039 USA; 2 Department of Pediatrics, University of Cincinnati College of Medicine, Cincinnati, Ohio USA

**Keywords:** Attention-deficit/hyperactivity disorder, Cognitive disengagement syndrome, Executive functions, Sluggish cognitive tempo

## Abstract

Cognitive disengagement syndrome (CDS) is a set of behaviors including mental confusion, excessive daydreaming, and slowed behavior and thinking that are strongly related to but distinct from attention-deficit/hyperactivity disorder (ADHD). A small body of research suggests that CDS may be associated with specific daily life executive functioning (EF) deficits in children, above and beyond ADHD symptoms. However, findings remain mixed, partially due to using smaller and ADHD-defined samples, mono-informant designs, and non-optimal measures of CDS. The current study examined multi-informant CDS and ADHD symptoms in relation to daily life EF in children (*N* = 263; ages 8–12 years; *M*±*SD*_age_ *=* 9.83 ± 1.42; 42.2% female). Caregivers and teachers completed measures assessing CDS, ADHD, and daily life EF. Multivariate regression analyses examined CDS and ADHD symptoms in relation to metacognitive and behavioral regulation EF domains. Across models with caregiver- and teacher-reported symptoms, ADHD-hyperactivity/impulsivity and ADHD-inattention symptoms were most consistently and strongly associated with behavioral and metacognitive EF domains, respectively. Nonetheless, even after accounting for ADHD symptoms, CDS was significantly associated with greater difficulties in metacognitive index subscales (Initiate, Working Memory, Plan/Organize, Organization of Materials, Monitor) and one behavioral regulation index subscale (Shift). This study provides evidence of the unique association between CDS and daily life EF, suggesting that CDS symptoms may be important to incorporate in models of EF. Future studies are needed to examine the interrelations of CDS and EF over time and in response to treatment.

Cognitive disengagement syndrome (CDS) includes a constellation of developmentally inappropriate cognitive and behavioral symptoms including excessive daydreaming, mental confusion, and hypoactivity (e.g., underactivity, slow movements; Becker et al., 2023). Initially conceptualized as a potential marker for a subset of individuals with attention-deficit/hyperactivity disorder (ADHD) predominantly inattentive presentation, dozens of studies have now demonstrated that CDS is a separate yet related construct from ADHD inattention (Becker et al., 2016, 2023). There is also now growing evidence for etiological, neuropsychological, temperament, and physiological factors that differentially relate to CDS and ADHD. Twin studies find CDS symptoms to be less heritable than ADHD symptoms (Moruzzi et al., 2014; Willcutt, 2020), suggesting there may be a more pronounced role of environmental factors in the etiological pathway to CDS behaviors (Moruzzi et al., 2014; Wiggs et al., 2025). In considering neurocognitive processes, there is evidence that CDS and ADHD-IN symptoms are differentially associated with slowed and poorer performance, respectively (Tamm et al., 2024), CDS symptoms are also associated with reduced working memory efficiency and faster inhibition speed, above and beyond ADHD inattention (Kofler et al., 2019), consistent with other studies linking CDS symptoms with increased behavioral inhibition system (BIS) sensitivity (Becker et al., 2013, 2018a, b) and higher sympathetic nervous system reactivity during a social stressor task (Becker & McQuade, 2020). Beyond these domains, whereas ADHD inattention is characterized by external distractibility, it has been theorized that CDS may be a behavioral manifestation of internal mentation or mind wandering (Becker & Barkley, 2021), with recent data supporting this possibility using self-report ratings (Fredrick & Becker, 2021; Fredrick et al., 2020), a mind wandering task (Wiggs et al., 2024), and neuroimaging studies linking CDS symptoms to atypical connectivity between task-positive and task-negative (i.e., default mode network) networks (Becker et al., 2025a; Camprodon-Rosanas et al., 2019). There is thus growing evidence across levels of analysis that CDS and ADHD are distinct syndromes, with likely different etiologies and underlying developmental and neurobiological processes.

As the empirical distinction between CDS and ADHD has been clarified alongside initial clues pointing to distinct neurocognitive and neurobiological factors, studies have shifted toward examining the unique daily life impairments linked to CDS, above and beyond ADHD. Evidence is accumulating that CDS symptoms contribute independently to functional impairment across multiple domains, including social, academic, and cognitive functioning (Becker, 2025; Becker et al., 2023; Kaçmaz et al., 2024). As the field continues to delineate the functional correlates and consequences of CDS, one area warranting further attention is executive functioning (EF), which is robustly linked to multiple domains of functioning (e.g., academic performance, socioemotional functioning; Spiegel et al., 2021; Zelazo, 2020).

There is clear evidence that ADHD dimensions are strongly associated with EF deficits, including daily life EF (e.g., observable, behavioral manifestations of EF in everyday situations; Antshel et al., 2013; Weyandt, 2009; Willcutt et al., 2005). Although laboratory-based tasks (performance-based measures) of EF provide valuable insights into the efficiency of specific cognitive processes in a structured environment, they may not fully capture how these difficulties manifest in real-world settings (Barkley & Fischer, 2011; Toplak et al., 2013). Rather, rating scales of EF (e.g., Behavior Rating Inventory of Executive Function [BRIEF]) provide information on one’s success in rational goal pursuit in an unstructured setting and may be a more representative, ecologically valid assessment of EF as it occurs in everyday life (Ten Eycke & Dewey, 2016; Toplak et al., 2013), such as behaviors observed at home or in school.

Two important EF domains for daily life include metacognition and behavioral regulation (Gioia et al., 2015). Metacognition represents one’s ability to cognitively self-manage tasks and monitor their performance, and includes one’s ability to initiate, plan, organize, and sustain future-oriented problem solving in working memory (i.e., ability to think about thinking). Children with metacognitive deficits may exhibit difficulties starting on schoolwork, challenges monitoring their progress while completing schoolwork or chores, or difficulties recognizing when they should ask a caregiver or teacher for assistance on tasks. Behavioral regulation is broadly defined as the ability to shift cognitive set (i.e., rules or strategies that guide how one approaches problems) and modulate emotions and behavior using inhibitory control, and it includes inhibit, shift, and emotional control (Gioia et al., 2015). Children with behavioral regulation deficits may have difficulties with emotion regulation, challenges switching from one task to the next, or difficulties stopping and thinking before acting.

Despite evidence that CDS is related to impairment in domains commonly affected in children and adolescents with ADHD (e.g., academic functioning, socioemotional functioning; Becker, 2025), relatively little is known about how CDS relates to EF, particularly as it manifests in daily life. In contrast, a far larger body of literature has examined ADHD dimensions in relation to daily life EF, consistently finding inattentive symptoms (ADHD-IN) to be related to metacognitive aspects of EF and hyperactive/impulsive symptoms (ADHD-HI) to be related to behavioral regulation domains of EF (Gioia et al., 2015; Jacobson et al., 2020; Molitor et al., 2019). However, most of these studies focused only on ADHD and did not also consider the potential role of CDS.

Among children and adolescents, the small body of research available suggests that CDS may be associated with specific daily EF domains above and beyond ADHD symptoms (Barkley, 2013; Becker & Langberg, 2014; Jimenez et al., 2015). Among children and adolescents (*N* = 76; ages 6–17), Jimenez and colleagues (2015) found CDS to be associated with numerous areas of caregiver-reported daily life EF, including emotional control, working memory, and plan/organize, when controlling for ADHD. Similarly, Capdevila-Brophy and colleagues (2014) found that children (ages 7–12) with both ADHD-IN and elevated CDS symptoms (*n* = 19) had greater caregiver-reported self-monitoring, working memory, and overall metacognitive EF deficits compared to children with ADHD-IN and low CDS symptoms (*n* = 68). Using a multi-informant design with both teacher and caregiver ratings in a sample of youth with ADHD (*N* = 52; ages 12–16), Becker and Langberg (2014) found aspects of CDS (e.g., slow, daydreamy, low initiation) to be uniquely associated with both behavior regulation and metacognitive domains of daily life EF. Notably, only the associations between caregiver-reported CDS slow symptoms and caregiver- and teacher-reported metacognitive symptoms remained significant when ADHD symptoms were included in the model.

In nationally representative samples spanning childhood and adolescence, youth with elevated symptoms of CDS (without elevated symptoms of ADHD) have been found to have greater caregiver-reported daily life EF deficits compared to youth without elevated symptoms of either CDS or ADHD, but fewer deficits compared to those with elevated symptoms of ADHD only (Barkley, 2013; Burns & Becker, 2021). The specific areas of daily life EF associated with CDS differed somewhat across these nationally representative samples; whereas both studies found CDS to be significantly associated with self-organization and problem-solving deficits, Burns and Becker (2021) found that CDS was also significantly associated with caregiver-reported deficits in emotion regulation.

Notably, the studies reviewed above found ADHD symptoms to be more consistently and strongly associated than CDS symptoms with daily life EF domains (Barkley, 2013; Becker & Langberg, 2014; Burns & Becker, 2021), yet individuals with both ADHD and CDS symptoms have even greater EF deficits compared to those with ADHD or CDS alone (Barkley, 2013; Burns & Becker, 2021). However, understanding of these associations remains unclear, partially due to limitations of previous studies including smaller and ADHD-defined samples (Becker & Langberg, 2014; Capdevila-Brophy et al., 2014) and mono-informant designs (Barkley, 2013; Burns & Becker, 2021; Jimenez et al., 2015). In addition, several previous studies used non-optimal measures of CDS, such as measures that include items that consistently load on an ADHD factor rather than a CDS factor (e.g., low motivation; Barkley, 2013; Becker & Langberg, 2014; Jimenez et al., 2015), which particularly clouds an ability to test for CDS-specific associations in relation to daily life EF.

Understanding the unique association between CDS and daily life EF is important in furthering our understanding of the impact of CDS symptoms beyond ADHD symptoms. Given that these EF deficits impact numerous daily life activities, understanding the association between CDS and EF could have implications for assessment and intervention planning. Clarifying the association between CDS, ADHD, and EF may inform targeted supports in school, home, and clinical settings for youth exhibiting high levels of CDS symptoms. Thus, the current study aims to further our understanding of the association between CDS and daily life EF. To test this aim, we used a multi-informant design in a sample of children recruited to include a full range of CDS symptom severity. Our sample included school-aged children (ages 8–12) as developmental models of EF indicate that most EFs are relatively mature by age 12 years (Anderson, 2002; see also Brocki & Bohlin, 2004) and EFs are strongly associated with academic outcomes during this developmental period (Spiegel et al., 2021).

## Methods

### Participants

Participants were 263 children (aged 8–12 years; *M*±*SD*_age_*=* 9.83 ± 1.42; 42.2% female; 75.3% White; 3.0% Hispanic/Latine). See Table 1 for sample characteristics. Of the 263 children, 168 (63.9%) met diagnostic criteria for ADHD based on the Kiddie Schedule for Affective Disorders and Schizophrenia for School-Age Children (K-SADS; Kaufman et al., 1997) conducted with the child’s caregiver.

**Table 1:** Sample characteristics (*N* = 263)

Age	M (SD) or *N* (%)
	9.83 (1.42)
Sex	
Female	111 (42.2%)
Male	152 (57.8%)
Race	
White	198 (75.3%)
Black	37 (14.1%)
Asian	3 (1.1%)
Multiracial	17 (6.5%)
Hispanic/Latine	8 (3.0%)
Family Income	
Up to $30,000	23 (8.7%)
$30,001 - $50,000	21 (8.0%)
$50,001 - $80,000	50 (19.0%)
$80,001 - $100,000	40 (15.2%)
$100,001 - $120,000	54 (20.5%)
Over $120,000	73 (27.85%)
Declined to answer	2 (0.8%)
Psychiatric diagnoses	
ADHD combined presentation	53 (20.2%)
ADHD predominantly inattentive presentation	113 (43.0%)
ADHD predominantly hyperactive-impulsive presentation	2 (0.8%)
Oppositional defiant disorder	21 (8.0%)
Conduct disorder	2 (0.8%)
Anxiety disorder	23 (8.7%)
Dysthymia	1 (0.4%)
Disruptive mood dysregulation disorder	0
ADHD stimulant medication use	82 (31.2%)

*ADHD *attention-deficit/hyperactivity disorder. For anxiety=presence of generalized anxiety disorder, social phobia, specific phobia, separation anxiety disorder, panic disorder, and/or posttraumatic stress disorder (PTSD)

### Procedures

All study procedures were approved by the Cincinnati Children’s Hospital Medical Center Institutional Review Board. To recruit children, flyers and emails were distributed throughout the community, local schools, and the medical center where the study was conducted. This study purposefully used a variety of recruitment materials to ensure a full range of CDS and attention difficulties were represented for dimensional-focused analyses (e.g., some materials included descriptors of CDS and ADHD symptoms, some materials omitted descriptors of attentional concerns). Interested families completed a phone screen assessing inclusion and exclusion criteria (e.g., age, diagnoses, medication status). Eligible participants were invited for an in-person evaluation during which informed consent, assent, and a release of information to gather teacher ratings were obtained. Exclusion criteria included taking non-stimulant psychotropic medications or being unwilling to wash out of stimulant medications for the assessment visit, IQ < 80, or history of epilepsy, seizures, or brain injury resulting in loss of consciousness. In addition, children diagnosed with psychosis, bipolar disorder, autism spectrum disorder, or obsessive-compulsive disorder, as reported by caregiver or as assessed by the K-SADS were excluded given the potential for these disorders to confound study aims.

### Measures

#### Demographic Characteristics and Medication Use

Caregivers completed a demographic questionnaire and medication form to gather the information reported in Table 1.

#### Clinical Interview

DSM-based psychopathology was assessed with the K-SADS (Kaufman et al., 1997), a widely-used semi-structured clinical interview. In the current study, caregivers were administered the ADHD, oppositional defiant disorder, conduct disorder, disruptive mood dysregulation disorder, anxiety disorder, and mood disorder modules, as well as the autism spectrum disorder screener items. In the current study, the K-SADS was exclusively used to describe psychiatric diagnoses of the sample (see Table 1). A participant was considered to meet criteria for a psychiatric diagnosis if they met DSM-5 diagnostic criteria as reported by their caregiver on the K-SADS. For example, a participant was determined to meet criteria for ADHD if caregivers endorsed (1) six or more symptoms of inattention and/or hyperactivity-impulsivity at clinically significant levels, (2) presence of symptoms prior to age 12, (3) presence of symptoms in two or more settings, (4) impairment related to symptoms, and (5) symptoms were not better explained by another disorder.

#### CDS and ADHD Symptoms

Caregivers and teachers completed the 9-item CDS, 9-item ADHD-inattention (ADHD-IN), and 9-item ADHD-hyperactivity/impulsivity (ADHD-HI) scales from the Child and Adolescent Disruptive Behavior Inventory (CADBI; Burns et al., 2014). Each item is rated on a six-point scale for the past month (0 = *Almost never*,* 1 = Seldom*,* 2 = Sometimes*,* 3 = Often*,* 4 = Very often*, and 5 = *Almost always*). The CADBI CDS symptoms have demonstrated separability from ADHD-IN and ADHD-HI symptoms in numerous samples (Belmar et al., 2017; Burns et al., 2017; Lee et al., 2014), in addition to strong internal consistency (del Mar Bernad et al., 2016; Lee et al., 2018), test-retest reliability (Burns et al., 2013; Servera et al., 2016), inter-rater reliability (Belmar et al., 2017), home-school invariance (Burns et al., 2017), and both concurrent (Fenollar Cortes et al., 2017; Khadka et al., 2016; Lee et al., 2014) and longitudinal (del Mar Bernad et al., 2014, 2016) external validity. In the current sample, reliability (i.e., alpha) for the caregiver and (teacher) CADBI CDS, ADHD-IN, and ADHD-HI scores were 0.95 (0.94), 0.96 (0.96), and 0.95 (0.93), respectively. Despite the psychometric strengths of the scale (Becker, 2021), there is not normative information for the CADBI; thus, we used the mean CDS, ADHD-IN, and ADHD-HI scores for each informant in the current study’s dimensional analyses, with higher scores indicating greater symptom severity.

#### Daily Life EF

Caregivers and teachers completed the Behavior Rating Inventory of Executive Function (BRIEF; Gioia et al., 2000), an 86-item questionnaire that assesses EF behaviors of children and adolescents in daily life (e.g., home and school environments). The BRIEF consists of two broad indices: the Metacognitive Index and the Behavioral Regulation Index.

The Metacognitive Index consists of five clinical subscales: (1) Initiate (i.e., beginning a task or activity; independently generating ideas, responses, or problem-solving strategies), (2) Working Memory (i.e., the capacity to hold information in mind of the purpose of completing a task), (3) Plan/Organize (i.e., ability to manage the current and future-oriented task demands, including ability to anticipate future events, set goals, and develop appropriate steps ahead of time to carry out a task or activity as well as the ability to bring order to information and to appreciate main ideas or key concepts when learning or communicating information), (4) Organization of Materials (i.e., orderliness of work, play, and storage spaces, as well as the manner in which children order or organize their world and belongings), and (5) Monitor (i.e., work checking habits including whether a child assesses their own performance during or shortly after finishing a task to ensure appropriate attainment of a goal as well as personal monitoring function such as whether a child keeps track of the effect their behavior has on others).

The Behavioral Regulation Index consists of three clinical subscales: (1) Inhibit (i.e., the ability to inhibit, resist, or not act on an impulse and the ability to stop one’s own behavior at the appropriate time), (2) Shift (i.e., the ability to move freely from one situation, activity, or aspect of a problem to another as the circumstances demand), and (3) Emotional Control (i.e., ability to modulate emotional responses) clinical scales.

Items are rated on a three-point scale (1 = *never*, 2 = *sometimes*, 3 = *often*) with higher scores indicated greater impairment. Consistent with a previous studies (Beer et al., 2011; Dekker et al., 2017; Smith et al., 2020), including research focused on CDS and daily life EF (Jimenez et al., 2015), total raw scores were used in the analyses and included both age and sex as covariates in the primary analyses.

Previous studies using the BRIEF have reported strong internal consistency (Becker & Langberg, 2014; Fournet et al., 2015; Gutiérrez-Colina et al., 2016; Huizinga & Smidts, 2010; Minnes et al., 2016; Modi et al., 2017; Park et al., 2016; Perez et al., 2017), inter-rater reliability (Rose & Holmbeck, 2007), test-retest reliability (Hughes et al., 2009; Huizinga & Smidts, 2010), and construct validity (Fournet et al., 2015; Huizinga & Smidts, 2010).

### Analyses

Analyses were conducted using Mplus statistical software (Muthén & Muthén, 1998–2017–2017) using the maximum likelihood with robust standard estimator (MLR) which is robust to nonnormality. Bivariate correlations were conducted to examine associations among demographic variables, psychopathology dimensions as measured by the CADBI, and daily life EF domains as measured by BRIEF. To reduce the number of models conducted, multivariate regression models were estimated to determine whether caregiver and teacher ratings of ADHD-IN, ADHD-HI, and CDS symptom dimensions were independently associated with daily life EF, with one model examining metacognitive indices and a second model examining behavioral regulation indices. Within both models, caregiver and teacher independent variables (ADHD-IN, ADHD-HI, and CDS) only predicted their respective metacognitive and behavioral regulation outcomes (both sources provided ratings on the same dependent variables); caregiver and teacher outcomes were allowed to correlate. Medication status (0 = not currently taking a prescribed stimulant medication, 1 = currently taking a prescribed stimulant medication), sex (0 = female, 1 = male), age, and income (range: 1 = under $30k to 7 = over $120k) were included in analyses as control covariates. Missing data was minimal (12.5% or less) across both analyses and was handled via maximum likelihood estimation (*N* = 263 was available for both models). A *p* < .05 (2-tail) defined statistical significance in all analyses.

## Results

### Bivariate Correlations

All psychopathology dimensions (CDS, ADHD-IN, ADHD-HI) were significantly positively correlated with each other (see Table 2, which also includes descriptive statistics of study variables). ADHD-IN and ADHD-HI severity were significantly positively correlated with each other for both caregiver- and teacher-reported symptoms (*r* = .64, *p* < .001 and *r* = .52, *p* < .001, respectively). Similarly, ADHD-IN and CDS severity were significantly positively correlated for both caregiver- and teacher-reported symptoms (*r* = .80, *p* < .001 and *r* = .81, *p* < .001, respectively). Despite strong associations, the magnitude of correlations are below what would be considered redundant (i.e., < 0.85; Brown, 2015; Little, 2024). Correlations between CDS and ADHD-HI severity were smaller in magnitude but still significant (caregiver *r* = .35, *p* < .001; teacher *r* = .18, *p* = .007).

**Table 2:** Bivariate correlations and descriptive statistics of study variables

Variable	1	2	3	4	5	6	7	8	9	10	11	12	13	14	15
1. Medication	--	0.00	0.05	− 0.03	0.08	0.11	0.18**	0.12	0.14*	0.03	0.07	0.08	0.12	0.10	0.17**
2. Income	0.00	--	0.05	0.07	− 0.28***	− 0.32***	− 0.15*	− 0.17**	− 0.11	− 0.10	− 0.27***	− 0.27***	− 0.30***	− 0.25***	− 0.21**
3. Sex	0.05	0.05	--	0.01	0.09	0.12	0.19**	0.22***	0.10	0.10	0.06	0.12	0.12	0.18**	0.18**
4. Age	− 0.03	0.07	0.01	--	0.03	− 0.08	− 0.13*	− 0.09	0.00	0.01	− 0.05	− 0.10	− 0.06	− 0.06	− 0.08
5. CDS	0.08	− 0.07	0.02	0.04	--	0.81***	0.18**	0.24***	0.36***	0.14*	0.75***	0.79***	0.75***	0.61***	0.58***
6. ADHD-IN	0.25***	− 0.10	0.03	− 0.09	0.80***	--	0.52***	0.54***	0.40***	0.25***	0.80***	0.86***	0.83***	0.76***	0.76***
7. ADHD-HI	0.28***	− 0.18**	0.08	− 0.24***	0.35***	0.64***	--	0.84***	0.42***	0.46***	0.33***	0.40***	0.34***	0.46***	0.63***
8. Inhibit	0.25***	− 0.15*	0.12	− 0.24***	0.31***	0.58***	0.85***	--	0.47***	0.53***	0.48***	0.48***	0.48***	0.52***	0.75***
9. Shift	0.14*	− 0.12*	0.07	− 0.09	0.53***	0.59***	0.51***	0.54***	--	0.74***	0.46***	0.45***	0.49***	0.41***	0.58***
10. Emotional Control	0.12	− 0.03	− 0.02	− 0.15*	0.44***	0.54***	0.52***	0.53***	0.68***	--	0.30***	0.23***	0.29***	0.27***	0.48***
11. Initiate	0.14*	− 0.20**	0.06	− 0.02	0.72***	0.80***	0.52***	0.50***	0.63***	0.53***	--	0.84***	0.84***	0.59***	0.78***
12. Working Memory	0.21***	− 0.18**	0.04	− 0.08	0.76***	0.89***	0.58***	0.54***	0.55***	0.49***	0.79***	--	0.87***	0.72***	0.75***
13. Plan/Organize	0.20***	− 0.20**	0.08	0.03	0.73***	0.86***	0.54***	0.51***	0.57***	0.47***	0.79***	0.85***	--	0.74***	0.74***
14. Organization of Materials	0.24***	− 0.14*	− 0.06	0.00	0.52***	0.73***	0.49***	0.44***	0.40***	0.39***	0.67***	0.71***	0.70***	--	0.66***
15. Monitor	0.24***	− 0.09	0.14*	− 0.07	0.58***	0.78***	0.63***	0.67***	0.60***	0.50***	0.75***	0.75***	0.79***	0.62***	--
*Caregiver Mean*	--	--	--	--	1.85	2.48	1.58	16.61	12.68	16.43	15.63	21.60	23.57	13.73	16.07
*Caregiver SD*	--	--	--	--	1.38	1.49	1.35	5.46	3.61	5.05	3.87	6.34	6.64	3.54	4.15
*Teacher Mean*	--	--	--	--	1.60	1.81	0.95	14.78	13.56	11.74	12.26	18.10	16.89	10.59	17.19
*Teacher SD*	--	--	--	--	1.31	1.49	1.11	5.33	3.92	3.94	4.32	6.20	5.61	4.03	5.18

*N* = 263. For executive functioning and psychopathology variables, caregiver-reported correlations are displayed below the diagonal and teacher-reported correlations are displayed above the diagonal. For medication use, 0 = not currently taking any prescribed stimulant medication, 1 = currently taking a prescribed stimulant medication. For sex, 0 = female, 1 = male. For income, an ordinal indicator was used with 1 = under $30,000, 7 = over $120,000. CDS=cognitive disengagement syndrome. ADHD-HI=attention-deficit/hyperactivity disorder hyperactivity-impulsivity. *ADHD-IN *attention-deficit/hyperactivity disorder inattention

*p<.05. **p<.01. ***p < .001

Regarding daily life EF domains, all caregiver-reported (*r*s = 0.39-0.85, all *p*s < 0.001) and teacher-reported (*r*s = 0.23–87, all *p*s < 0.001) daily life EF domains were significant positively associated with each other. Caregiver-reported daily life EF domains were significantly associated with caregiver-reported CDS severity (*r*s = 0.31-0.76, all *p*s < 0.001), ADHD-IN severity (*r*s = 0.54-0.89, all *p*s < 0.001), and ADHD-HI severity (*r*s = 0.49-0.85, all *p*s < 0.001). Similarly, all teacher-reported daily life EF domains were significantly associated with teacher-reported CDS severity (*r*s = 0.39-0.85, all *p*s < 0.001), though associations were lower for behavior regulation index subscales (*r*s = 0.14-0.36, all *p*s < 0.05) compared to metacognitive index subscales (*r*s = 0.58-0.79, all *p*s < 0.001). In addition, teacher-reported daily life EF domains were significantly associated with teacher-reported ADHD-IN severity (*r*s = 0.25-0.86, all *p*s < 0.001) and ADHD-HI severity (*r*s = 0.33-0.84, all *p*s < 0.001).

### Multivariate Regression Analyses Examining Metacognitive Indices

Results for models examining the total Metacognitive Index and Metacognitive subscales can be found in Table 3. Caregiver-reported CDS severity was not significantly associated with the total Metacognitive Index (β = 0.08, *p* = .07), but teacher-reported CDS severity was significantly associated with total Metacognitive Index (β = 0.23, *p* < .001). Results for models examining the Metacognitive subscales showed caregiver ratings of CDS severity were significantly positively associated with Initiate (β = 0.24, *p* < .001), Working Memory (β = 0.13, *p* = .008), and Plan/Organize (β = 0.12, *p* = .027), and significantly negatively associated with Organization of Materials (β = -0.17, *p* = .019); teacher ratings of CDS severity were significantly positively associated with Initiate (β = 0.35, *p* < .001), Working Memory (β = 0.33, *p* < .001), Plan/Organize (β = 0.21, *p* = .002), and Monitor (β = 0.16, *p* = .036). Caregiver ratings of ADHD-IN were significantly positively associated with Initiate (β = 0.57, *p* < .001), Working Memory (β = 0.75, *p* < .001), Plan/Organize (β = 0.73, *p* < .001), Organization of Materials (β = 0.87, *p* < .001), and Monitor (β = 0.64, *p* < .001); teacher ratings of ADHD-IN severity were also significantly positively associated with Initiate (β = 0.50, *p* < .001), Working Memory (β = 0.56, *p* < .001), Plan/Organize (β = 0.68, *p* < .001), Organization of Materials (β = 0.69, *p* < .001) and Monitor (β = 0.44, *p* < .001). Finally, caregiver ratings of ADHD-HI severity were significantly positively associated with Monitor (β = 0.23, *p* < .001) only; teacher ratings of ADHD-HI severity also were only significantly positively associated with Monitor (β = 0.36, *p* < .001).

**Table 3:** Multivariate regression analyses examining ADHD dimensions and CDS symptoms in relation to BRIEF metacognitive index outcomes

	Initiate				Working Memory				Plan/Organize				Organization of Materials				Monitor				Metacognitive Index		
	β	SE	*p*		β	SE	*p*		β	SE	*p*		β	SE	*p*		β	SE	*p*		β	SE	*p*
CR Symptoms	*R*^2^ = 0.68, *p*<.001				*R*^2^ = 0.81, *p*<.001				*R*^2^ = 0.76, *p*<.001				*R*^2^ = 0.57, *p*<.001				*R*^2^ = 0.65, *p*<.001				*R*^2^ = 0.85, *p*<.001		
Sex	0.04	0.04	0.28		0.02	0.03	0.51		0.05	0.03	0.07		− 0.09*	0.04	0.03		0.10**	0.04	0.006		0.03	0.02	0.17
Age	0.04	0.04	0.27		− 0.00	0.03	0.89		0.10**	0.03	0.002		0.09*	0.04	0.04		0.04	0.04	0.30		0.06*	0.03	0.017
Income	− 0.12**	0.04	0.001		− 0.09**	0.03	0.001		− 0.12***	0.03	< 0.001		− 0.07	0.04	0.12		0.01	0.04	0.90		− 0.09***	0.03	< 0.001
Medication	− 0.04	0.04	0.30		0.01	0.03	0.86		0.01	0.03	0.83		0.04	0.04	0.34		0.01	0.04	0.75		0.01	0.03	0.85
CDS	0.24***	0.06	< 0.001		0.13**	0.05	0.008		0.12*	0.06	0.027		− 0.17*	0.07	0.019		− 0.01	0.07	0.93		0.08	0.04	0.07
ADHD-IN	0.57***	0.07	< 0.001		0.75***	0.06	< 0.001		0.73***	0.06	< 0.001		0.87***	0.08	< 0.001		0.64***	0.08	< 0.001		0.81***	0.05	< 0.001
ADHD-HI	0.07	0.05	0.18		0.03	0.04	0.42		0.03	0.04	0.55		− 0.01	0.06	0.94		0.23***	0.05	< 0.001		0.07*	0.04	0.033
TR Symptoms	*R*^2^ = 0.67, *p*<.001				*R*^2^ = 0.77, *p*<.001				*R*^2^ = 0.72, *p*<.001				*R*^2^ = 0.60, *p*<.001				*R*^2^ = 0.66, *p*<.001				*R*^2^ = 0.82, *p*<.001		
Sex	− 0.04	0.04	0.30		0.02	0.03	0.56		0.03	0.04	0.45		0.07	0.04	0.09		0.04	0.04	0.31		0.03	0.03	0.36
Age	− 0.01	0.04	0.74		− 0.06	0.03	0.09		− 0.02	0.04	0.59		0.01	0.04	0.91		− 0.00	0.04	0.96		− 0.02	0.03	0.46
Income	− 0.01	0.04	0.75		0.01	0.03	0.73		− 0.03	0.04	0.37		− 0.01	0.04	0.84		0.03	0.04	0.53		− 0.00	0.03	0.98
Medication	− 0.01	0.04	0.73		− 0.02	0.03	0.58		0.04	0.04	0.26		0.00	0.04	0.99		0.05	0.04	0.23		0.01	0.03	0.64
CDS	0.35***	0.07	< 0.001		0.33***	0.06	< 0.001		0.21**	0.07	0.002		0.02	0.08	0.82		0.16*	0.07	0.036		0.23***	0.05	< 0.001
ADHD-IN	0.50***	0.08	< 0.001		0.56***	0.07	< 0.001		0.68***	0.08	< 0.001		0.69***	0.09	< 0.001		0.44***	0.09	< 0.001		0.67***	0.06	< 0.001
ADHD-HI	0.01	0.05	0.81		0.05	0.04	0.27		− 0.07	0.05	0.17		0.09	0.06	0.11		0.36***	0.05	< 0.001		0.09*	0.04	0.023

For medication, 0 = not currently taking any prescribed stimulant medication, 1 = currently taking a prescribed stimulant medication. For sex, 0 = female, 1 = male. For income, an ordinal indicator was used with 1 = under $30,000, 7 = over $120,000. *ADHD-HI *attention-deficit/hyperactivity disorder hyperactivity-impulsivity, *ADHD-IN *attention-deficit/hyperactivity disorder inattention, *CDS *cognitive disengagement syndrome, *CR *caregiver-report, *TR *teacher-report

*p<.05. **p<.01. ***p < .001

### Multivariate Regression Analyses Examining Behavioral Regulation Indices

Results for models examining the total Behavioral Regulation Index and Behavioral Regulation subscales can be found in Table 4. Neither caregiver- nor teacher-reported CDS severity was significantly associated with the total Behavioral Regulation Index (caregiver: β = 0.13, *p* = .06; teacher: β = 0.15, *p* = .08). Results for models examining the Behavioral Regulation subscales showed caregiver (β = 0.28, *p* = .001) and teacher (β = 0.35, *p* = .001) ratings of CDS severity were significantly positively associated with Shift only. Caregiver ratings of ADHD-IN severity were significantly positively associated with Inhibit (β = 0.15, *p* = .039) and Emotional Control (β = 0.21, *p* = .048), whereas teacher ADHD-IN severity was significantly positively associated with Inhibit only (β = 0.19, *p* = .012). Caregiver ADHD-HI severity was significantly positively associated with Inhibit (β = 0.77, *p* < .001), Shift (β = 0.29, *p* < .001), and Emotional Control (β = 0.34, *p* < .001); teacher ADHD-HI severity was also significantly positively associated with Inhibit (β = 0.74, *p* < .001), Shift (β = 0.40, *p* < .001), and Emotional Control (β = 0.51, *p* < .001).

**Table 4:** Multivariate regression analyses examining ADHD dimensions and CDS symptoms in relation to BRIEF behavior regulation index outcomes

	Inhibit				Shift				Emotional Control				Behavior Regulation Index		
	β	SE	*p*		β	SE	*p*		β	SE	*p*		β	SE	*p*
CR Symptoms	*R*^2^ = 0.73, *p*<.001				*R*^2^ = 0.41, *p*<.001				*R*^2^ = 0.37, *p*<.001				*R*^2^ = 0.64, *p*<.001		
Sex	0.06	0.03	0.08		0.04	0.05	0.38		− 0.05	0.05	0.29		0.02	0.04	0.69
Age	− 0.04	0.03	0.28		− 0.01	0.05	0.80		− 0.06	0.05	0.23		− 0.04	0.04	0.25
Income	− 0.01	0.03	0.84		− 0.03	0.05	0.53		0.07	0.05	0.16		0.02	0.04	0.63
Medication	0.01	0.03	0.87		− 0.01	0.05	0.83		− 0.04	0.05	0.41		− 0.02	0.04	0.64
CDS	− 0.08	0.06	0.16		0.28**	0.09	0.001		0.17	0.09	0.06		0.13	0.07	0.06
ADHD-IN	0.15*	0.07	0.039		0.19	0.11	0.07		0.21*	0.11	0.048		0.19*	0.08	0.023
ADHD-HI	0.77***	0.04	< 0.001		0.29***	0.07	< 0.001		0.34***	0.07	< 0.001		0.59***	0.05	< 0.001
TR Symptoms	*R*^2^ = 0.72, *p*<.001				*R*^2^ = 0.27, *p*<.001				*R*^2^ = 0.22, *p*<.001				*R*^2^ = 0.54, *p*<.001		
Sex	0.06	0.04	0.08		− 0.01	0.06	0.92		0.00	0.06	0.99		0.03	0.05	0.57
Age	0.03	0.04	0.36		0.04	0.06	0.52		0.07	0.06	0.24		0.05	0.05	0.25
Income	− 0.02	0.04	0.52		0.02	0.06	0.73		− 0.03	0.06	0.62		− 0.01	0.05	0.79
Medication	− 0.03	0.04	0.34		0.05	0.06	0.36		− 0.06	0.06	0.33		− 0.02	0.05	0.71
CDS	− 0.06	0.07	0.40		0.35**	0.11	0.001		0.14	0.11	0.21		0.15	0.09	0.08
ADHD-IN	0.19*	0.08	0.012		− 0.08	0.13	0.55		− 0.13	0.13	0.33		0.02	0.10	0.86
ADHD-HI	0.74***	0.04	< 0.001		0.40***	0.07	< 0.001		0.51***	0.07	< 0.001		0.68***	0.05	< 0.001

For medication, 0 = not currently taking any prescribed stimulant medication, 1 = currently taking a prescribed stimulant medication. For sex, 0 = female, 1 = male. For income, an ordinal indicator was used with 1 = under $30,000, 7 = over $120,000. *ADHD-HI *attention-deficit/hyperactivity disorder hyperactivity-impulsivity, *ADHD-IN *attention-deficit/hyperactivity disorder inattention, *CDS *cognitive disengagement syndrome, *CR *caregiver-report, *TR *teacher-report

*p<.05. **p<.01. ***p < .001

## Discussion

Using a multi-informant design, the current study examined the associations between severity of CDS and ADHD symptoms with daily life EF among school-aged youth. This study extends previous work in several important ways, including use of a measure of CDS with strong psychometric properties (including discriminant validity from ADHD), multiple informants (i.e., caregiver and teacher), and a non-ADHD-referred sample. When examining associations at the total index level, we did not find significant associations between CDS severity and caregiver- and teacher-reported Behavior Regulation or caregiver-reported Metacognitive Index above and beyond ADHD severity. However, when examining daily life EF at the subscale level, we found that CDS symptom severity was independently associated with multiple aspects of daily life EF above and beyond ADHD symptom severity, including an aspect of Behavioral Regulation (i.e., Shift), as well as Metacognitive domains (i.e., Initiate, Working Memory, Plan/Organize, Organization of Materials, Monitor). Taken together, these findings suggest that CDS symptoms may be associated with a different pattern of EF difficulties that is not reflected in the overall index scores, highlighting the importance of evaluating daily life EF with greater specificity.

Consistent with previous research (Gioia et al., 2015; Jacobson et al., 2020; Molitor et al., 2019), ADHD symptoms were more consistently and strongly associated than CDS symptoms with daily life EF domains. Notably, inattentive symptoms were strongly associated with metacognitive subscales and hyperactive/impulsive symptoms were strongly associated with behavioral regulation subscales. Given the strong associations between ADHD symptoms and BRIEF scores, the fact that CDS symptoms were nevertheless independently associated with numerous domains of daily life EF domains bolsters confidence in our findings that CDS symptoms are uniquely associated with a specific set of daily life EF deficits.

Teacher-reported, but not caregiver-reported, CDS severity was significantly associated with the total Metacognitive Index. Despite caregiver-reported CDS severity not being significantly associated with the total Metacognitive Index, both caregiver- and teacher-reported CDS severity were significantly associated with several Metacognitive indices of daily life EF, which is consistent with previous research using the BRIEF (Becker & Langberg, 2014; Capdevila-Brophy et al., 2014). In this study, the specific Metacognitive indices associated with CDS severity varied by informant, and the magnitude of associations between CDS symptoms and these Metacognitive subscales were generally larger for teacher-report compared to caregiver-report, providing further evidence that daily life EF is context-specific. Specifically, both caregiver- and teacher-reported CDS symptoms were significantly associated with higher Initiate, Working Memory, and Plan/Organize scores above and beyond ADHD-IN and ADHD-HI symptoms. CDS symptoms were not significantly associated with teacher-reported Organization of Materials, but were significantly negatively associated with caregiver-reported Organization of Materials. Overall, these results are consistent with previous research that has found CDS to be associated with Working Memory (Capdevila-Brophy et al., 2014; Jimenez et al., 2015) and Plan/Organize (Jimenez et al., 2015) EF ratings, but diverges from previous work that did not find significant associations with the Initiate subscale. Whereas previous research did not find a significant association between CDS and caregiver-reported Organization of Materials scores when controlling for ADHD (Jimenez et al., 2015), we found a significant negative association between CDS symptoms and caregiver-reported Organization of Materials scores.

The association between CDS symptoms and Metacognitive aspects of daily life EF may be in part due to slowed cognitive processing and working memory deficits, which have been linked to CDS symptoms in prior studies using performance-based neuropsychological assessments (Kofler et al., 2019; Tamm et al., 2024). In addition, the manifestation of CDS symptoms in both the home and school setting may impact metacognitive aspects of daily life EF. For example, hypoactivity symptoms such as slow behavior may impact how quickly one can start a task (Initiate), mental confusion symptoms (e.g., thinking gets mixed up; easily confused) may impact one’s ability to generate ideas, problem-solve independently (Initiate), or plan and organize their thoughts and actions (Plan/Organize), and daydreaming symptoms (e.g., gets lost in own thoughts; appears lost in a fog) could impede one’s ability to hold information in their mind for the purpose of completing a task (Working Memory).

Notably, teacher-reported CDS symptoms, but not caregiver-reported symptoms, were significantly associated with higher Monitor subscale scores, which assesses work-checking habits (i.e., whether a child assesses their own performance during or shortly after finishing a task to ensure appropriate attainment of a goal) and personal monitoring (i.e., whether a child keeps track of the effect his or her behavior has on others). Previous research has indicated that CDS is significantly associated with academic impairment, including difficulties with study skills and learning strategies (Fredrick & Becker, 2023b), as well as social impairment, including social withdrawal, lower social engagement, and deficits in picking up on social cues (Fredrick & Becker, 2023a). Teachers may have more opportunities to observe the Monitor difficulties given different academic and social demands in the school environment (Van Tetering & Jolles, 2017), which may be why we found associations between CDS symptom severity and teacher-reported but not caregiver-reported Monitor scales. Our findings differ from Capdevila-Brophy and colleagues (2014), who found children with ADHD-IN and high caregiver-reported CDS symptoms to have clinically significant elevations on Self-Monitoring. These mixed findings underscore the importance of using multi-informant approaches and including ADHD symptomatology in studies examining CDS symptoms in relation to daily life EF.

Previous studies have not found CDS to be significantly related to Behavioral Regulation at the broad index level (Becker & Langberg, 2014; Duncan et al., 2019). However, some studies have found CDS to be significantly associated with aspects of behavioral regulation, including emotional control (Jimenez et al., 2015), emotion regulation (Burns & Becker, 2021), and self-restraint (Barkley, 2013). In contrast, we found that both caregiver- and teacher-reported CDS symptoms were significantly associated with a different aspect of Behavioral Regulation (i.e., Shift). When controlling for ADHD symptoms, CDS symptoms were significantly associated with higher Shift scores, defined as “the ability to move freely from one situation, activity, or aspect of a problem to another as the circumstances demand” (Gioia et al., 2000, p. 18). Notably, the magnitude of the association between CDS symptoms and Shift was similar to the magnitude of the association between ADHD-HI symptoms and Shift. It may be that aspects of CDS (e.g., slow moving, thinking is slow) may be interpreted by others as a child being unwilling to move on from one task to another. In addition, the Shift subscale enquires about susceptibility to perseveration (e.g., “Thinks too much about the same topic”), which may reflect the daydreaming aspects of CDS. The Shift subscale also assesses difficulties adapting to new situations (e.g., “Has trouble getting used to new situations (classes, groups, friends)”). Children with high CDS severity may be observed to have difficulties adapting in social situations, given that CDS is associated with increased social withdrawal (Becker et al., 2025b; Fredrick & Becker, 2023a; Inci Izmir et al., 2024) and fear or discomfort and avoidance of social situations (Becker et al., 2013).

### Theoretical and Clinical Implications

Findings from this study have several theoretical implications. Despite well-established links between ADHD and EF – as measured using daily life scales or neuropsychological tests – our findings indicate that CDS severity may add additional predictive power in a subset of EF domains. These findings add to a growing body of evidence pointing to CDS as having an important role in the long-standing interest to understand the heterogeneity of ADHD (Becker, 2025; Nigg, 2026). Generally, youth with co-occurring elevations in both CDS and ADHD symptoms have worse daily life EF functioning than youth with elevations in either syndrome alone (Barkley, 2013; Burns & Becker, 2021), which may suggest that our findings point to clinical severity in relation to daily life EF. However, it is also important to note that there were differential findings across EF domains when CDS, ADHD-IN, and ADHD-HI were examined as individual predictors, and so a psychopathological severity hypothesis does not fully explain our findings. In addition, a recent study in a nationally representative sample of Spanish youth found children in a CDS-only group to have strong differential validity from all three ADHD presentations, including ADHD predominantly inattentive presentation, with 51% of children (ages 5–10 years) and 33% of adolescents (ages 11–16) with clinically-elevated CDS symptoms *not* qualifying for clinical elevations in *any* ADHD presentation (Burns et al., 2025). Our findings also build on prior studies showing CDS and ADHD to be differentially associated with speed vs. accuracy on neurocognitive tasks (Tamm et al., 2024), as well as in regard to prospectively assessed internalizing vs. externalizing psychopathologies (Becker et al., 2018a; del Mar Bernad et al., 2016). Considered together, these findings raise the possibility that CDS may ultimately be best conceptualized as a separate syndrome from ADHD, or a specifier for ADHD that improves clinical understanding and prediction (Becker, 2025; Nigg, 2026). To inform these possibilities, rigorous multi-method, developmentally-informed studies are needed to test whether there are distinct etiological processes (e.g., environmental stressors, mind wandering, atypical brain connectivity) that contribute to the presence of CDS (vs. ADHD) behaviors, which give rise to both distinct and shared daily life EF deficits. Equifinality, the possibility that diverse pathways lead to the same outcome, is a core tenant of developmental psychopathology (Cicchetti & Rogosch, 1996) and may be important when examining CDS and ADHD, as there may be distinct developmental processes that lead to similar outcomes, at least for a subset of EF domains.

The current findings that CDS severity is associated with Metacognitive aspects of daily life EF also have potentially important implications for assessment and intervention planning for youth. Further understanding of the association between symptoms of CDS, ADHD, and daily life EF can provide better support for adolescents who experience both CDS and ADHD symptoms and/or a high level of CDS symptoms. Mixed findings on the Monitor subscale underscore the context-specific nature of daily life EF deficits, indicating clinicians should prioritize the approach of obtaining information from multiple informants that may see a child in different settings. Given that CDS severity is associated with metacognitive deficits, interventions may need to focus on supports that scaffold internalized cognitive control (e.g., structured routines, external reminders, attention prompts) rather than externalizing behavior management (e.g., token economies). Children with CDS symptoms that have daily life EF deficits may benefit from existing interventions that focus on academic success for children, especially those that target deficits with self-monitoring and initiating behaviors. Future studies are needed to examine the interrelations of CDS and EF over time and in response to treatment.

### Strengths, Limitations, and Future Directions

Strengths of this study include the use of multiple informants for symptoms of CDS, ADHD, and daily life EF deficits, in addition to the community-based sample of children presenting with a range of CDS symptomatology. Additionally, the study utilized psychometrically-supported measures of CDS, ADHD, and daily life EF.

Certain limitations of this study are important to note and should be considered in future research. Given our study did not aim to recruit a national or community-based sample, as well as the lack of norms for CDS using the CADBI measure, we prioritized dimensional approaches in analyzing our data. Future research using modern measures of CDS that have norms should consider conducting categorical analyses to further explore potential group differences between those with CDS only, ADHD only, CDS and ADHD, and typically developing children. In addition, we focused on ADHD and CDS symptoms given previous research with mixed findings and our use of a larger sample, strong measure of CDS, and multiple informants. Future research should consider the impact of other psychiatric comorbidities on daily life EF. The cross-sectional nature of this study precludes causal claims or the ability to establish temporal precedence, and longitudinal research is needed to evaluate the potential impact of CDS symptoms on the development or exacerbation of daily life EF among children as they progress into adolescence and adulthood. Additionally, we relied on caregiver- and teacher-report of psychopathology symptoms and EF deficits, and previous work suggests that youth self-report of CDS symptoms may also be valuable (Becker, 2021). Future research should incorporate both daily life and observational measures of EF to get a comprehensive picture of CDS-related EF deficits. Caregiver and teacher ratings were likely completed based on how they typically observed the child and given that almost a third of our sample was prescribed stimulant medications, these ratings may limit our understanding of symptoms without medication. In addition, youth taking psychotropic medications other than stimulants were excluded from this study; therefore, these results may not extend to children taking non-stimulant medications. Finally, the sample was predominantly White, of higher socioeconomic status, included only school-aged children, and a large proportion meeting criteria for ADHD which may limit generalizability. Future research should aim to prioritize studies in more diverse and representative samples, including considerations of factors that may impact how CDS manifests in daily life EF.

## Conclusion

The current study adds to the growing literature on CDS by being the first to examine the unique associations between CDS and ADHD symptom dimensions and daily life EF using multiple informants (i.e., caregiver and teacher) and a supported measure of CDS. When examining daily life EF at the subscale level, CDS symptoms were significantly associated with both caregiver- and teacher-reported domains of daily life EF, above and beyond ADHD symptoms. Overall, our findings suggest that symptoms of CDS may be associated with different patterns of EF difficulties compared to ADHD symptoms. Future research should consider CDS in models of EF.

## Data Availability

Data preparation and descriptive statistics were done in SPSS 28. Data and code are available from the corresponding author upon reasonable request and execution of a data use agreement.
